# Elastomer Characterization Method for Trapped Rubber Processing

**DOI:** 10.3390/polym12030686

**Published:** 2020-03-19

**Authors:** Pooria Khalili, Thomas Boulanger, Brina J. Blinzler

**Affiliations:** Division of Material and Computational Mechanics, Department of Industrial and Materials Science, Chalmers University of Technology, SE-412 96 Göteborg, Sweden; pooriak@chalmers.se (P.K.); th.boulanger13@gmail.com (T.B.)

**Keywords:** composites, processing, elastomers, reinforced polymer composites, autoclave alternative, TRP

## Abstract

The increasing high-volume demand for polymer matrix composites (PMCs) brings into focus the need for autoclave alternative processing. Trapped rubber processing (TRP) of PMCs is a method capable of achieving high pressures during polymer matrix composite processing by utilizing thermally induced volume change of a nearly incompressible material inside a closed cavity mold. Recent advances in rubber materials and computational technology have made this processing technique more attractive. Elastomers can be doped with nanoparticles to increase thermal conductivity and this can be further tailored for local variations in thermal conductivity for TRP. In addition, recent advances in computer processing allow for simulation of coupled thermomechanical processes for full part modeling. This study presents a method of experimentally characterizing prospective rubber materials. The experiments are designed to characterize the dynamic in situ change in temperature, the dynamic change in volume, and the resulting real-time change in surface pressure. The material characterization is specifically designed to minimize the number and difficulty of experimental tests while fully capturing the rubber behavior for the TRP scenario. The experimental characterization was developed to provide the necessary data for accurate thermomechanical material models of nearly incompressible elastomeric polymers for use in TRP virtual design and optimization.

## 1. Introduction

As the demand for carbon fiber reinforced polymers (CFRPs) increases, the need for autoclave alternatives for high performance composite processing that allows faster throughput while maintaining performance becomes more pronounced. Current autoclave processing of composites relies on polymer compaction through a combination of vacuum bagging and pressurization. The autoclave is heated in a way that increases temperature in the air that is transferred to the surface of the part. This procedure is somewhat inefficient and can take a matter of hours for the part to go through the full cure cycle [1,2]. Additionally, autoclaves are costly to both acquire and maintain and must be large enough to accommodate for the largest part produced [3]. Pressurized bladder molding (PBM) is another alternative processing technique where the composite preform is placed in a hard cavity mold and pressurized with an internal bladder. This allows for the cure cycle to be reduced to under an hour for thin parts but can be susceptible to bladder rupture and is typically only used for small parts due to the cost of the exterior tooling [4,5]. 

There exists an industry need for autoclave alternatives to high performance composite processing that allows for faster throughput without giving up performance. In the mid-1970s, a number of polymeric elastomers were developed for TRP [6,7,8,9], but the research stalled due to the low thermal conductivity of the materials and the disconnect between change in temperature and change in pressure for complex shapes. Over-pressurization is a common initial problem when using a trial-and-error based process design methodology. It is clear that numerical process models are required for TRP processing design. A well characterized rubber material model can then be used in conjunction with existing process modeling methods [10]. Recent advances in technology have made the TRP processing technique achievable for complex shapes and high-volume production. Extensive research in the computer electronics industry has developed a number of elastomers with high thermal conductivity. This increase in thermal conductivity is generally achieved by using nanoscale metallic additives [11]. 

It is well known that the through-thickness degree of cure or crystallinity gradients cause non-thermoelastic residual stresses during PMC manufacturing [10]. Through-thickness cure gradients are exacerbated primarily by two mechanisms. One is the rate of thermal loading and the other is the thickness of the composite preform. High throughput automated PMC manufacturing can require high-temperature curing, but sharp distortions are intensified by increasing the processing temperature range [12,13]. In-plane residual stresses can be further intensified by increasing the thickness of the composite preform [14,15,16,17,18]. It is more efficient for thick parts, to processes the component in a single cycle, but typically multiple cycles are used to processes parts greater than the recommended thickness ranges due to the severity of cure gradient, residual stresses, and other phenomena [18]. Nano-additives can be exploited to customize the thermal conductivity of the TRP material [19] and mitigate these thermal gradient effects. In this way, sections of the TRP unit in contact with thicker sections of the composite preform can be designed with a comparatively higher conductivity to those other sections in contact with thinner composite preform. Additionally, this can potentially eliminate locally under-cured or over-cured areas of the structure. There has also been investigation into copolymer formulations that combine the hyperplastic properties of one polymer with the high thermal conductivity properties of another polymer [20]. TRP in general is an ideal candidate for nanoscale optimization of multifunctional mechanical and thermal properties. 

**Figure 1:**
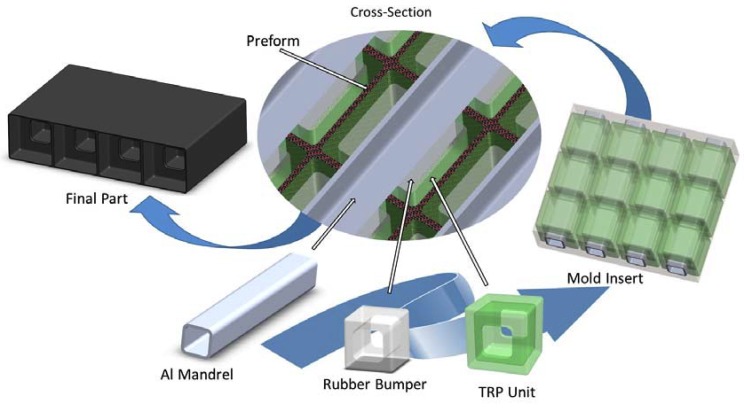
Advanced trapped rubber molding process.

Once optimal thermal conductivity is achieved, then the remaining question is how to link the temperature change with changes in pressure. In this paper, a solution is proposed. This coupled thermomechanical process can be simulated with finite element analysis [21]. An experimental characterization method has been designed to capture the volumetric change in pressure via a series of material tests. These tests are specifically designed to characterize the in-situ change in temperature, the change in volume of the nearly incompressible elastomeric material and the volumetric change in surface pressure on the exterior surface of the TRP unit (Figure 1). 

One of the benefits of advanced trapped rubber processing is that there is no need for an autoclave, which can be costly both to acquire and to maintain [3]. Also, in contrast to PBM there is no need for a pneumatic system, which is prone to rupture and reduces the complexity of the design [4]. Future advanced TRP will also eliminate the need for ovens because the heating technology can be incorporated directly into the rubber material at discrete locations where it is needed for the best cure profile. The parts made using this processing technique are highly consolidated, which is desirable for high performance composites. This process also lends itself more easily to robotic manufacturing when compared with autoclave processing and PBM. Considering both the reduction in energy consumption during processing and a reduction in labor the development of a robust TRP method could lead to more efficient and sustainable reinforced polymer composite manufacturing. In this paper, a straightforward experimental characterization method for TRP materials is proposed. The method limits deviatoric strains via geometric constraints and captures in situ pressure and temperature transients in ranges similar to those required for processing thermoset polymer matrix composites.

## 2. Materials and Methods 

**Figure 2:**
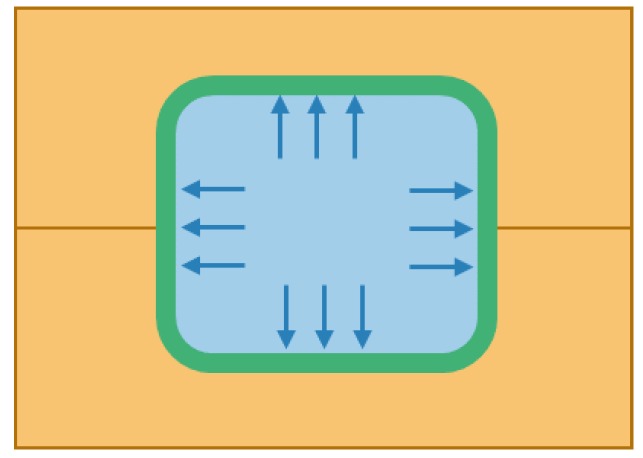
Cross section of trapped rubber processing (TRP).

TRP allows more design freedom with more advanced shapes and less risk of processing failure while maintaining the possibility for custom distributions of pressures, custom distributions of temperatures, and therefore high-quality consolidation and curing. Simple TRP is shown in Figure 2. Here, the outer clamshell mold is shown in yellow, the rubber is blue and the composite preform is green. The rubber expands due to the temperature change and this imparts a uniform pressure on the composite preform. 

An advanced TRP molding technique (Figure 1) can be developed by isolating the rubber material that has the large volumetric change. In the illustration in Figure 1, the cavity mold, aluminum mandrel, and rubber bumper have relatively low coefficients of thermal expansion. While the TRP unit is tailored to expand for cure pressurization in alignment with the resin system employed in composite preform. As illustrated here, this process also is amenable to robotic manufacturing. 

### 2.1. Specimen Development

**Figure 3:**
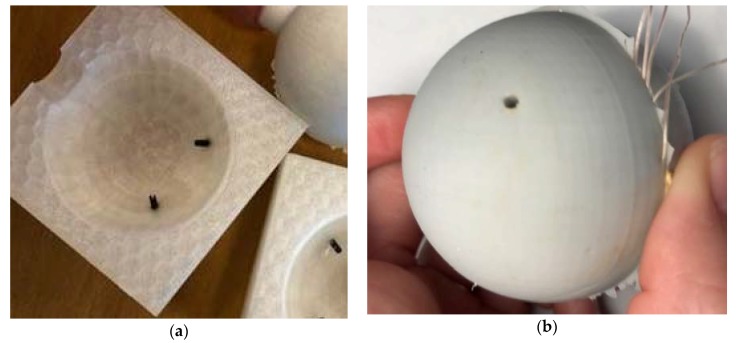
3D printed mold and resulting rubber sphere molded concentrically over a steel sphere: (**a**) 3D printed rubber mold; (**b**) Demolded rubber assembly.

The experimental setup is designed and conducted to capture the volumetric change and resulting pressure via a series of tests. These tests include: the change in temperature, the change in volume, and the change in surface pressure. The temperature and surface pressure test setup consist of aluminum molds with an internal steel sphere and the rubber material in a concentric hollow sphere. The thermal expansion of the aluminum molds and the steel sphere can be calculated easily during the cure cycle and thereby simplify the characterization of the rubber material. The hollow rubber sphere (Figure 3) is molded to have an outer diameter of 60.0 mm and an inner diameter of 50.00 mm at room temperature. The two-part silicone rubber is poured into the rubber manufacturing mold with the 50.00 mm diameter steel sphere suspended in the center. The resulting concentric hollow sphere is a test specimen with a uniform thickness for straight forward characterization.

A CS25 condensation cure silicone rubber was purchased from Easy Composites Ltd. (Stoke-on-Trent, United Kingdom) The combined rubber and catalyst were grey in color, had the viscosity of 20,000–26,000 mPa at ambient temperature and had 1.04–1.14 g/cm^3^ density. The solid steel spheres were purchased from Spekuma company and had the weight of 510 g measuring 50.00 mm in diameter [22]. Loctite hysol 9466 A&B adhesive was used to bond four spring pins in radially oriented holes in the 3-D printed PLA molds. The thermocouples used for embedding were TC-TT-K-40-36 purchased from Omega Engineering Limited. The processing time for the adhesive was 60 min, and the adhesive had the viscosity of 35 Pa·s and possessed the tensile strength of 32 MPa. Mold release wax was applied to the inner surface of the PLA molds and the A-staged rubber mixture was degassed for 3 min at a vacuum of 26.6 millibar before pouring into the mold. The mold was vibrated during pouring to mitigate bubbles forming on the reversed curvature portion of the top of the mold. 

**Figure 4:**
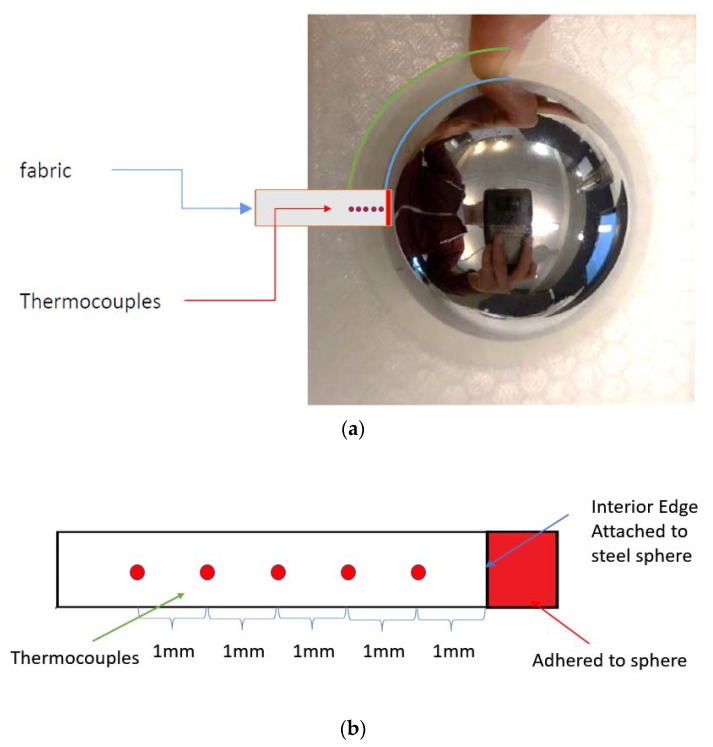
Position of the thermocouples in reference to the sample geometry: (**a**) View of the rubber mold, steel sphere, fabric and thermocouple placement location; (**b**) Detail view of thermocouple placement on fabric.

For the in-situ temperature acquisition, five TC-TT-K-40-369 thermocouples (TC) were embedded in the silicone rubber. These TCs have a diameter of 0.08 mm. In order to embed the TCs as evenly as possible through the thickness of the rubber during the casting process, the TCs were glued to a thin piece of fabric that was suspended in the rubber manufacturing mold prior to the addition of the liquid rubber. One side of the fabric was adhered to the steel sphere and the other side was temporarily taped to the exterior of the mold. During this process, the fabric had to be kept taut to mitigate sagging and maintain the through thickness location of the TCs. The TCs were placed on the fabric as shown in Figure 4. The TCs had to be perpendicular to the mold surface and parallel to each other, to ensure even acquisition of the local in-situ temperature. The TC wires were then threaded through the mold line and labeled according to through-thickness position. 

It is extremely difficult to place the TCs exactly 1 mm from each other per the target location. The exact locations were measured after the adhesive cured at 1.33, 2.31, 2.99, 3.70, and 4.65 mm measured from the interior edge attached to the steel sphere (Figure 4). Additionally, the exposed edges of the TCs were wrapped in tape to limit rubber adhesion outside the desired sphere shape. 

### 2.2. Rubber Specimen Molding

Two-part PLA molds with spherical cavity of 60.0 mm in diameter were 3D-printed for use in casting the silicon rubber around the suspended steel ball. ZYYXpro 3D printer was used to extrude the proPLA filament for mold manufacturing sold by ZYYX Labs, AB [23]. Two small holes were also designed at the bottom of each 3D-printed PLA molds to be able to suspend the steel ball during the rubber casting by gluing (Loctite hysol 9466 A&B adhesive) four steel spring pins into the holes and subsequently resting the ball on the four spring pins. 

The stochiometric ratio of silicon rubber to catalyst (100 to 5 parts by weight) was used, and the mix was degassed for the period of 3 min. Then the silicon rubber was poured gently into the three printed PLA molds with the suspended steel ball. The system was left to cure for 24 h before demolding and the whole demolded assembly had the weight of 560 g.

### 2.3. Temperature Change

**Figure 5:**
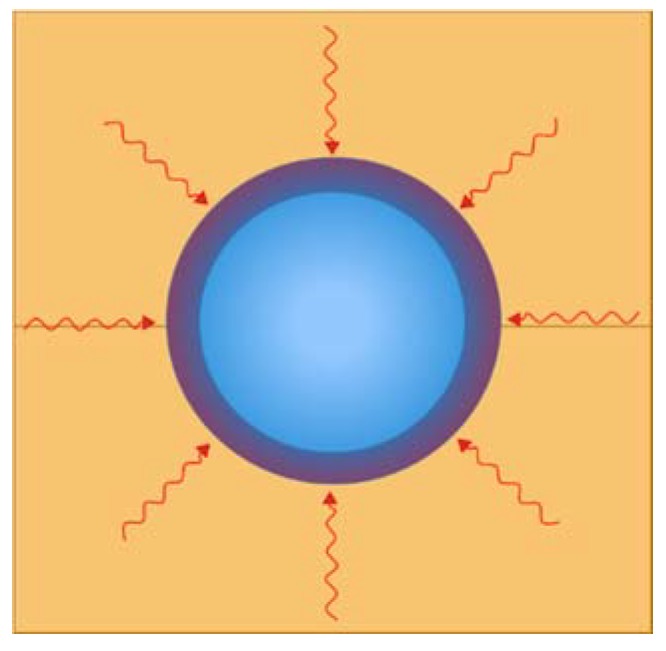
Temperature change test schematic.

The thermal transient was evaluated by placing the hollow rubber sphere in a two-part cavity mold, Figure 5. The rubber is effectively trapped between the outer surface of the steel sphere and the inner surface of the mold. Heat is then applied from the outside of the mold via flat aluminum plates under pressure containing electrical heating elements. TCs applied to the external mold are used to monitor the temperature change over time. Additionally, five TCs are distributed though the thickness of the rubber to capture the in-situ thermal transient with high accuracy. These TCs are molded into the rubber material to achieve an accurate through thickness heating map in real-time per the locations in Section 2.1. The model is constructed to effectively represent the test apparatus including the full mold for simulation [21,24]. Due to the high thermal conductivity of the aluminum cavity mold, the interior temperature at the rubber surface can be assumed uniform. The simulations confirm this and show less than 0.0001 °C of difference in temperature on the inner cavity surface of the aluminum mold during the full simulation [21].

**Figure 6:**
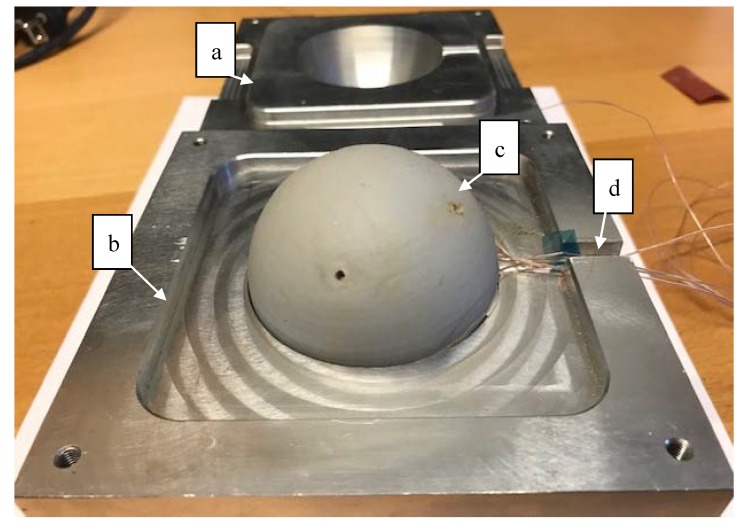
Temperature change test set-up: (**a**) top mold; (**b**) bottom mold; (**c**) rubber specimen; (**d**) data acquisition channel.

For this test setup TC-TT-K-40-36 TCs are used (Figure 6). These fine gauge TCs can be placed inside the mold without distorting the silicone rubber or the mold surfaces. The TCs are connected to a standard National Instruments™ data acquisition system cDAQ-9174 with 9212 Thermocouple C Series Module [25]. 

Adhering the TCs to fabric for internal placement within the silicone rubber resulted in the most robust method. However, with an additional material, there is always the risk for insulating the TCs. In order to assess this risk, a test was performed to evaluate the potential insulting impact of two different adhesives; LOCTITE super glue precision and LOCTITE EA 9466 which is a two-part epoxy glue. The two adhesives were then compared with a bare TC, without glue, as a control. 

### 2.4. Volume Change

Thermal expansion of CS25 silicone rubber was measured on a Q400 TA instrument, thermomechanical analyzer (TMA) [26] in standard mode which is recommended for most solid materials. A preload of 0.01 N was applied to the sample covered by two layers of aluminum foils on top and bottom to ensure good contact or remove slack. An applied force of 0.05 N was considered during the measurement and the heating rate was set to 5 °C/min. The thickness of the sample was approximately 2.88 mm and the temperature ramped from 30 °C to 200 °C. The temperature range was initially chosen to be in the compatible zone with generic aerospace grade epoxy resin systems such as Hexcel HexPly® M21E/IMA [27,28,29]. M21E resin used for structural PMCs, has a complex formulation that includes three types of epoxy, a hardener, and two thermoplastic polymers and has been involved in a number of studies [30]. The curing properties are well documented, and this makes M21E an ideal benchmarking material for processing characterization procedures. The sample was held at 30 °C for 5 min at the beginning of the test. The data from the second heating cycle was used to plot the relevant figures. The volume change test can be conducted using a TMA device [31]. TMA can be used to detect both linear and volumetric material expansion due to increasing temperature. This type of testing can be used to find more precise coefficients of thermal expansion for the silicone rubber material [31]. 

### 2.5. Pressure Change

**Figure 7:**
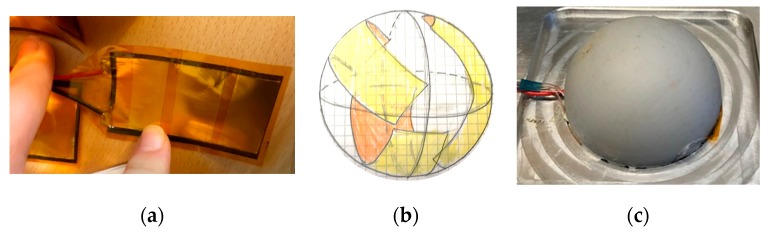
Sensing system: (**a**) at style dynamic pressure sensor; (**b**) Pressure sensor distribution inside the mold; (**c**) Open mold with rubber sample and pressure sensor.

The pressure transient is captured by placing the hollow rubber sphere in a two-part cavity mold. Heat is then applied from the outside of the mold. A series of real-time pressure sensing devices are embedded in the mold to obtain pressure on the outer surface of the rubber. In this study, the Tactilus® Free Form Sensor System produced by Sensor Products, Inc. [32] is used. Using this system real-time surface pressure can be obtained inside the mold coincident with the temperature readings. Three dynamic pressure sensors are used (Figure 7a), and the data acquisition software collects min, max, and average surface pressure on the sensors collectively. An individual sensor was measured at approximately 3.0 × 6.0 × 0.27 mm and is made of a flexible material that conforms to the shape of the mold. A schematic of the sensor placement inside the mold can be seen in Figure 7b. The pressure sensors are extremely thin and can be place inside the mold without significantly changing the surface contact (Figure 7c). 

The temperature range was initially chosen to be in the compatible zone with generic aerospace grade epoxy resin systems such as Hexcel HexPly® M21E/IMA [29]. However, as the pressure limit for the sensors was designed to be 20 bar, and there was no gap in the test specimen-mold configuration in order to prevent uncontrolled convection, neither the composite press nor the pressure sensors were able to handle the pressures predicted from the preliminary simulations [21]. Thus, the temperature range was modified to a reasonable pressure range, where the whole assembly was cooled to 5 °C and abruptly brought to room temperature (22.3 °C).

### 2.6. Processing Mold Design

**Figure 8:**
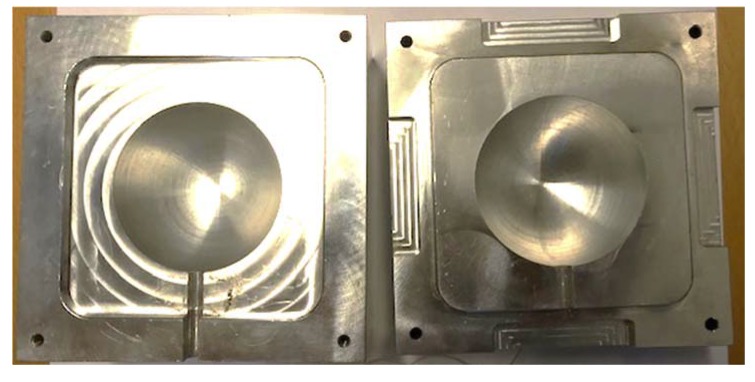
Aluminum processing mold.

The final clamshell mold was designed to replicate a situation that would be observed in trapped rubber processing of polymer matrix composites components (Figure 8). Aluminum 6061-T6 was selected for the mold due to both its machinability, high thermal conductivity, and thermal stability at the temperatures and pressures typically used to process thermoset composite components [33]. The cavity was designed to have an inner diameter of 60.00 mm and a surface roughness of around 0.80 micrometers. Each half of the clamshell mold is 60.00 mm tall. This large thickness ensures even heat flux on the outer surface of the silicone rubber independent of the surface-to-surface proximity of the inner cavity and outer surface. A small channel is included in the mold for the TCs and pressure sensor wires. The channel is designed so that there is no stress on the wires during testing. Mold alignment is achieved by use of the locking square shape that is recessed on the lower part and embossed on the upper part. This alignment shape has a tolerance of 50 micrometers laterally. Additionally, the processing mold is designed to have a high surface flatness, ensuring good thermal conductivity when used for plate-based heating in a composite press or similar processing apparatus. 

## 3. Results

The experiments were conducted in order to characterize the rubber material for development of a simulation-based tool for predicting local pressures on complex geometries cured using trapped rubber processing. The tests have been simplified as much as possible to limit the number of parameters required for characterization and limit convective heat transfer [12,16]. 

### 3.1. Preliminary Temperature Change Test

**Figure 9:**
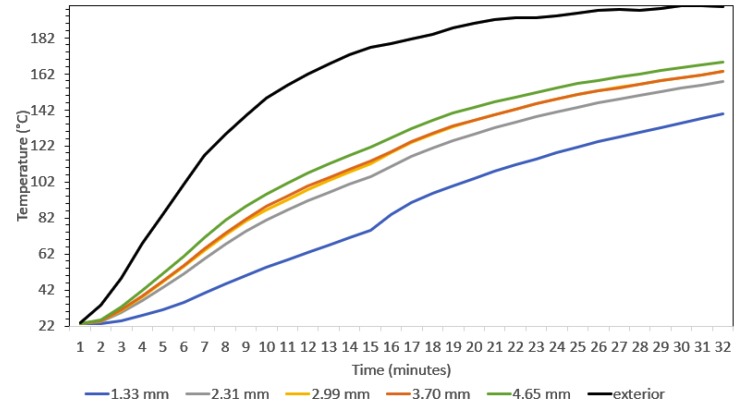
Thermal transient in-situ temperature distribution results.

For the adhesive insulation test, the three TCs were placed inside the oven and the temperature was increased from 22 °C to 200 °C at an approximate ramp rate of 12.5 °C/min while real-time temperature data was collected. The three TCs collected similar temperatures. However, there was a 12.7 and 17.8 °C/min lag in the LOCTITE super glue precision and LOCTITE EA 9466, respectively. After 3 min at 200 °C all TCs converged to within 2.0 °C. Because the LOCTITE super glue precision had a slightly lower effect on the resulting temperature reading, it was selected for sample preparation. A preliminary temperature change test was conducted to ensure that the embedded TCs were behaving as expected. This initial test was conducted without the mold by placing the molded silicone rubber sphere with the embedded TCs and steel sphere in the oven and increasing the temperature. The rubber test sample was placed inside the oven and the temperature was ramped from 22 °C to 200 °C at a ramp rate of approximately 7.5 °C/min. The results are shown in Figure 9. Here, the black curve is the temperature on the outer surface of the rubber, green is the TC that is nearest the outer surface of the rubber and dark blue is the TC closest to the interior steel sphere. It was noted that the oven temperature did not ramp smoothly, but instead resulted in some stepping as the heating elements cycled on and off. Additionally, once the oven reaches 150 °C the heating begins to slow and there is an obvious lag in interior readings. Furthermore, there is a significant heat sink experienced by the TC near the steel sphere. This was expected; however, mitigating this effect would make the test setup more efficient. 

### 3.2. Volume Change Test

**Figure 10:**
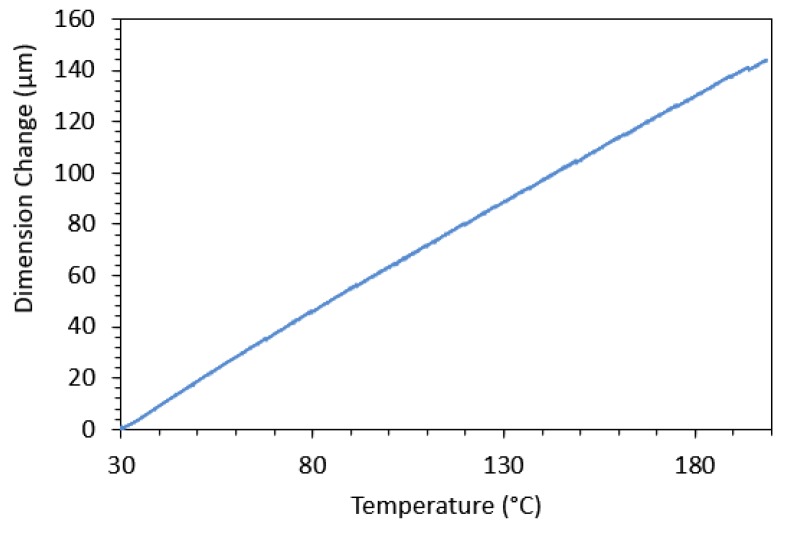
Thermal expansion of CS25 silicone rubber, dimension change vs. temperature.

The volume change was performed on the cured rubber and Figure 10 shows a plot displaying measured dimension change for the rubber. No volume change parameter was available from the datasheet of the supplier. The temperature was increased from 30 °C to 200 °C from the second heating cycle on the TMA instrument. It was found that the change of dimension is almost linear with a slope of 0.9 µm/°C. This demonstrates the consistent sensitivity of rubber to the heat at the temperature range tests. 

### 3.3. Temperature–Volume–Pressure Change Test

The temperature-volume-pressure change test consisted of assembling the silicone rubber sample with the embedded TCs and steel sphere inside the aluminum mold with the pressure sensors at the surface between the rubber and the aluminum. The exterior of the mold was then heated slowly while both real-time temperature and pressure data were acquired. The objective of this test was to analyze the pressure at the interface between the rubber and the outer mold surface during the heat up and dwell cycles similar to those used in composite autoclave processing. The center consists of a solid steel sphere with a rubber hollow sphere molded around it and then the two-part aluminum clamshell mold is placed around the rubber. As described in detail in Section 2.5, the pressure transducer system is placed between the aluminum mold and the rubber surface. Three pressure transducers are placed at the rubber–aluminum interface.

In order to establish a baseline for the rubber pressure due to full clamp up of the mold. Pressure data was taken at the initial temperature. It was also established that there is approximately 2 kPa of noise in the sensors. Additionally, there was a pressure peak that occurred during clamp up. This takes approximately 15 s to dissipate. After the pressure stabilized, the baseline pressure at the initial test temperature was approximately 76 kPa (760 millibar) at 5 °C. 

**Figure 11:**
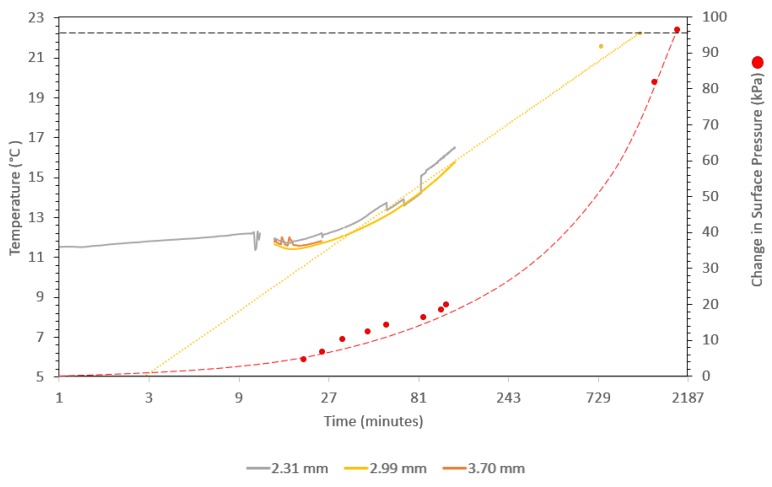
Full processing test results; left axis temperature of the embedded thermocouples (grey, orange, and yellow), yellow dashed line is the trend line of the thermocouple readings, the dashed black line is the external air temperature, and the right axis displays surface pressure between the rubber and aluminum clamshell mold, time is displayed in log base 3.

To mitigate convection, the initial rubber test sample and the clamshell mold have a tight fit. Because of this, the pressure-temperature test ranges were selected to observe the change in pressure and temperature change induced by moving the apparatus from an outdoor to an indoor environment. In this test, the apparatus is cooled to approximately 5 °C and placed in a room at 22.3 °C while observing the changes. The full apparatus, aluminum mold with rubber sample, embedded TCs, and pressure sensors, were chilled for 6 h at a temperature of approximately 5 °C. Once chilled, the apparatus was moved to an area where the air temperature measured 22.3 °C. It took approximately 1930 min for the full sample to reach equilibrium at 22.3 °C and an internal surface pressure of 96.5 kPa. During this time, embedded temperature readings and surface pressure were recorded. The results can be seen in Figure 11. The pressure is normalized such that it is zero at the initial test start time. The real-time acquisition of in-situ temperature and pressure data demonstrates the viability of this approach as a method for characterization of a hyper-elastic materials for developing a virtual processing model for TRP design. Based on the data here and the coefficient of thermal expansion measured in Section 3.2, it is relatively straight forward to calibrate a computational material model. 

## 4. Discussion

Overall, the characterization method for TRP materials presented here is thorough and can be used somewhat independently of a full understanding of the resin material vitrification process. The overall geometry and data acquisition system worked as expected, and the experimental data acquired can be directly used to model a TRP material in a process modeling simulation. However, there were a couple of issues encountered and these should be followed up with future studies. 

Concerning the specimen geometry and the assembly configuration for testing the following was observed and discovered during the study. The geometry of the processing mold was found to be adequate for testing and constrained the rubber in a way that prevents deviatoric strains which allows for easier characterization for modeling. The initial materials performed as expected in this test. For future testing, processing silicone rubbers should be used and molded in a similar way in order to obtain a hollow sphere of rubber around a solid sphere. The steel sphere at the center of the specimen was straight forward to align during molding, but did absorb a considerable amount of heat. The characterization method could be improved by using a hollow rigid sphere made of a material that could be used in polymer matrix composite processing, in order to reduce the time for the assembly to stabilize thermally. 

One of the assumptions made in this characterization study is that the coefficient of thermal expansion of the rubber is roughly linear above freezing. While this has been verified for the general casting silicone rubber used for this initial study, material tailored for TRP will not exhibit a linear behavior. In order to capture the in-situ readings of the cure profile for TRP, a method must be developed to either account for heat transfer in an air gap (which is not representative of PMC processing) or develop a material that behaves like a B-staged composite preform in durometer and thermal conductivity. 

With respect to the data acquisition and sensors, the following was observed and discovered during the study. While the TC embedding process is repeatable and relatively easy to perform, there is still some insulation experienced due to the glue used. It could be good to find an adhesive that works the same mechanically, but has a higher thermal conductivity or a similar thermal conductivity to that of the TRP material. 

The pressure sensor placement was quite difficult. In future studies, the use of a temporary spray adhesive might be beneficial in fixing the upper sensors positions. Additionally, the current sensor pattern adds approximately 0.54 mm to the diameter of the rubber sphere. Because the rubber sphere was molded to exactly fit the cavity, this produced a slight initial pressure. It may be beneficial for future rubber specimens to be molded with a planned 0.54 mm gap, although this could lead to shear deformation and other problems and the effects should be studied further. 

It can be observed in Figure 11 that the thermocouple readings are showing a higher plateau than expected at around 11.5 °C. This is likely due to the steel sphere not coming fully to equilibrium at 5 °C. This indicates that more than 6 h is needed for the full assembly to come to equilibrium at the cold temperature. 

Regarding the results, the coefficient of thermal expansion measured is in the range for silicone elastomers, but the pressures are slightly lower than initially predicted. The measured coefficient of thermal expansion of the CS25 silicone rubber was 286 × 10^−6^/K. This is within the range for typical silicone rubbers reported by AZoM as 250 to 300 × 10^−6^/K [34]. Pressures in processing can be extremely high due to the large Poisson’s ratio of the TRP material [7,31]. However, while the pressures observed here did exhibit the expected exponential progression with increasing temperature [31], the magnitude was not as great as predicted in the calculations [21]. Further simulation of the test configuration needs to be completed which includes both the compliance of the bolts and the possibility of gaps caused by the arrangement of the pressure sensors. 

In closing, this initial characterization procedure using a generic, inexpensive molding silicone rubber, demonstrates a straightforward approach to capturing the most important material properties for virtual design and optimization of TRP.

## 5. Conclusions

A study of TRP characterization for composite processing simulation has been presented here. In the broader landscape of high-performance composite processing, there is an open need for autoclave alternatives that allow for faster throughput without sacrificing material performance. TRP allows more design freedom with more advanced shapes and less risk of processing failure while maintaining the possibility for custom distributions of pressures and custom distributions of temperatures, and therefore high-quality consolidation and curing. There is still some work remaining in order to bring this research to a standardized characterization for simulating TRP. However, this study provides an initial method for a characterization framework aimed at filling the gap in specialized rubber material data required for TRP modeling. This should make it feasible to simulate processing for new and potentially complex or more automated polymer matrix composite component manufacturing processes. 

Most importantly, the work presented here demonstrates the viability of this straightforward characterization approach for determining physical parameters required for developing a computational material model for simulating TRP. With only two types of testing—a high precision measurement of the volume change to determine the coefficient of thermal expansion, and a combination of in-situ temperature and pressure measurements—a computational material model can be developed. This approach can be used in the future to aid in the development of tailored nanoparticle conductivity optimization of the trapped elastomeric material.
